# COVID-19 pandemic and capital markets: the role of government responses

**DOI:** 10.1007/s11573-022-01103-x

**Published:** 2022-07-07

**Authors:** Christian Beer, Janine Maniora, Christiane Pott

**Affiliations:** 1 TU Dortmund University, Dortmund, Germany; 2 Heinrich-Heine-University Düsseldorf, Düsseldorf, Germany

**Keywords:** COVID-19, Government policies, Investor sentiment, Capital market, Sales revenue, Behavioral finance, G11, G18, G41, I18, C23

## Abstract

This paper analyzes the moderation effect of government responses on the impact of the COVID-19 pandemic, proxied by the daily growth in COVID-19 cases and deaths, on the capital market, i.e., the S&P 500 firm’s daily returns. Using the Oxford COVID-19 Government Response Tracker, we monitor 16 daily indicators for government actions across the fields of containment and closure, economic support, and health for 180 countries in the period from January 1, 2020 to March 15, 2021. We find that government responses mitigate the negative stock market impact and that investors’ sentiment is sensitive to a firm’s country-specific revenue exposure to COVID-19. Our findings indicate that the mitigation effect is stronger for firms that are highly exposed to COVID-19 on the sales side. In more detail, containment and closure policies and economic support mitigate negative stock market impacts, while health system policies support further declines. For firms with high revenue exposure to COVID-19, the mitigation effect is stronger for government economic support and health system initiatives. Containment and closure policies do not mitigate stock price declines due to growing COVID-19 case numbers. Our results hold even after estimating the spread of the pandemic with an epidemiological standard model, namely, the susceptible-infectious-recovered model.

## Introduction

The COVID-19 pandemic infected stock markets worldwide. Recent studies show negative investor reactions to be the strongest since the Spanish Flu of 1918 (Zhang et al. 2020). For instance, the S&P 500 index dropped by more than 30% compared to its all-time high on January 16, 2020. Several economic and social lockdowns caused unexpected, exogenous shocks that provoked a high level of uncertainty in the world’s capital markets (Baker et al. 2020; Zhang et al. 2020). A large amount of unfiltered negative news shaped investors’ sentiment and expectations about the pandemic’s economic impact, reinforced market pessimism and triggered investor overreactions (Alexakis et al. 2021; Liu et al. 2021; Salisu and Vo 2020). In a recent study, Erdem (2020) reveals that the pandemic has a significant negative impact on a country's stock market index, with the growth in COVID-19 cases causing a three times larger decline in index prices than fatalities.

**Fig. 1:**
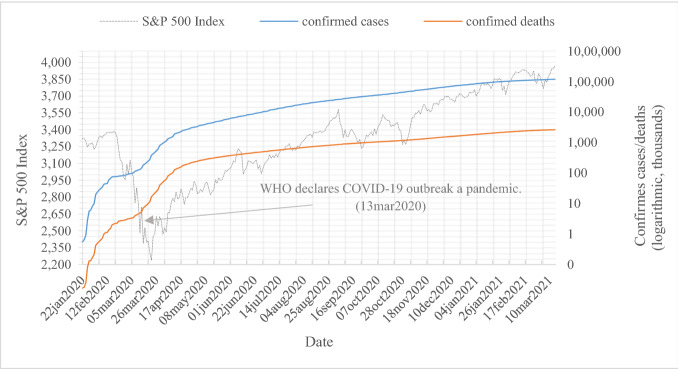
S&P 500 Index and globally confirmed COVID-19 cases and deaths (logarithmic). This figure shows the S&P 500 stock market index and logarithmic global confirmed COVID-19 cases and deaths for our observation period from January 1, 2020 to March 15, 2021. S&P 500 index data are derived from Thomson Reuters Datastream, and case and death data are obtained from the *European Centre for Disease Prevention and Control (ECDC)*

However, after the World Health Organization (WHO) pronounced COVID-19 a pandemic on March 13, 2020, it took only 26 days for the S&P 500 index to recover to its preannouncement value. Remarkably, another 158 days later, on August 18, 2020, the index again surpassed its all-time high from January 16, 2020. Figure 1 illustrates an overlay of the S&P 500 stock market index and the logarithmic growth of global confirmed COVID-19 cases and deaths for an observation period from January 1, 2020 to March 15, 2021.

What triggered investors to regain optimism rapidly, with the number of cases and deaths still rising? Recent studies on the capital market effects caused by the pandemic argue that government responses to contain the spread of the disease may play an important role in shaping investor sentiment during the pandemic (Alexakis et al. 2021; Hale et al. 2021; Salisu and Vo 2020). However, there is a lack of research on the relevance of government responses to COVID-19 for investor sentiment and capital markets. Only a few studies exist that, at an early stage, either discuss country-level macroeconomic impacts of government initiatives to contain the spread of the disease (e.g., Alexakis et al. 2021; Zaremba et al. 2020) or the impact of COVID-19 on investor sentiment (e.g., Jiang et al. 2021; Sun et al. 2021).

Research calls for studies that investigate the impact of government responses to COVID-19, differentiating government responses by their aim and scope to reveal diverse impacts on investor behavior and capital markets (e.g., Goodell 2020; Hale et al. 2021). Undoubtedly, firms have a deep interest in how to react to a pandemic crisis depending on government policies, leading to the development of communication strategies by all groups of stakeholders. Existing research does not consider that investors of multinational companies (MNCs) are forced to incorporate the policies of multiple governments into their trading decisions. Research thus far does not consider that trust in governments impacts investor sentiment and trading behavior during the pandemic. As the literature provides evidence that external shocks (e.g., terrorist attacks) significantly reduce investors’ trust (Lesmeister et al. 2018), government countermeasures to COVID-19 that reduce investors’ uncertainty can be assumed to positively affect trust and mitigate stock price declines. In addition, recent studies show that firm-specific characteristics may serve as moderators of COVID-19-associated declines in stock prices. For example, Ding et al. (2021) find that firms that are highly exposed to supply chain disruptions exhibit greater declines in returns. However, no study exists that analyzes associations with a firm’s sales side, i.e., sales revenues, by considering that investors can potentially evaluate the COVID-19 situation in other countries, where a large portion of a firm’s revenue is realized.

Our paper addresses this research gap. We argue that government responses influence investor sentiment, leading to diverse moderating effects on the association between the growth rates of COVID-19 cases, the number of deaths and stock market reactions. While restrictive policies may negatively influence investors’ sentiment, increase pessimism, and trigger market overreactions, investors may also appreciate supportive efforts by governments, adjust their perceptions about market development, and, in consequence, positively adjust their investment decisions. Hence, governments may be able to actively reduce uncertainty, increase trust among investors and indirectly affect the stock market. We expect this two-sided effect to be an important driver of rapid stock market recovery during the pandemic.

**Fig. 2:**
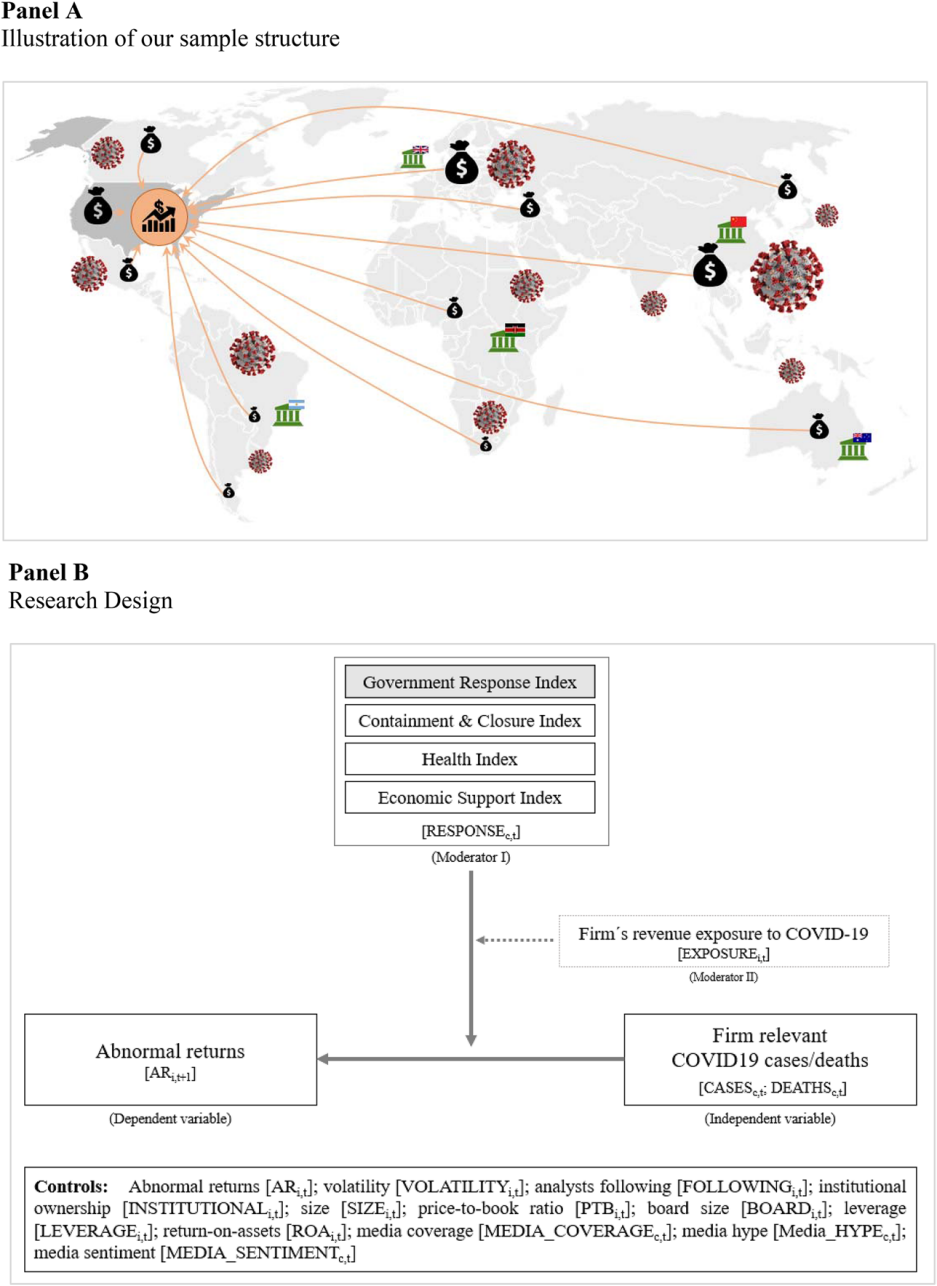
**A** Illustration of our sample structure. This graphic illustrates the structure of our sample. We use the S&P 500 firm-specific daily abnormal stock returns to proxy for U.S. capital market effects. Each firm realizes sales revenues at different levels in various countries, symbolized by the money bag. At the same time, these countries are exposed to the COVID-19 pandemic with different severeness over time, symbolized by the virus pictogram. To contain the spread of the disease and to mitigate the economic and social impacts of the pandemic, the countries’ governments respond with country-specific actions and policies, symbolized by the government house pictogram. **B** Research Design. This figure represents the design of our main regression model: COVID-19 cases and deaths of firm-relevant countries serve as the independent variables. Abnormal returns, measured as adjusted abnormal logarithmic returns using a market model, where expected returns are estimated with market betas using the firm’s daily stock returns, and the S&P 500 index returns of the respective period, is the dependent variable. We employ two moderators to test for interactions with our COVID-19 proxies. First, a set of three government response indices, calculated from 16 indicators covering three major fields of government policies, i.e., containment and closure, health system, and economic support, tracked by the Oxford University’s Government Response Tracker (OxCGRT), including a summarizing index to reflect the entirety of government responses. Second, a firm’s specific revenue exposure to COVID-19 was calculated by weighting the country-specific sales revenues of our sample firms with the country’s growth rates of COVID-19 cases and deaths per million

We build on the research of investor sentiment to explore the impact of the responses of 180 governments on the relationship between the stock prices of S&P 500 firms and the growth rates of COVID-19 cases and deaths in the period from January 1, 2020 to March 15, 2021. Specifically, we analyze 16 indicators covering three major fields of government policies, i.e., containment and closure, health system, and economic support, tracked by Oxford University’s Government Response Tracker (OxCGRT). We use country-specific revenues of each S&P 500 firm to solely assign government responses of firm-relevant countries to a firm and, in a second model, to reveal whether investors are aware of and react differently to a firm’s direct revenue exposure to COVID-19. Please see Fig. 2 for an illustration of our sample structure and our research design.

Results reveal that aggregated government responses mitigate the decline of stock returns due to both rising COVID-19 cases and deaths. Governments’ actions in the fields of containment and closure as well as economic support are most likely appreciated by investors and mitigate negative stock market impacts caused by the pandemic. In contrast, government actions concerning a country’s health system provoke further declines in abnormal stock returns. One reason could be the absence of initiatives that support health systems, e.g., widespread testing or vaccination campaigns, in the initial period of the pandemic. In general, health system initiatives are delayed compared to other government responses, causing uncertainty among investors. The mitigating effect of government responses in the field of economic support is stronger for firms that are highly exposed to COVID-19 on the sales side. We fail to find such effect for containment and closure policies, indicating that they are not strong enough for investors to adjust their pessimistic views on market development. Our results are relevant for firms in anticipating and strategically managing investor relations and in actively demanding government interventions. They are further valuable for governments in discussing the economic costs and benefits of their responses to pandemics. Moreover, our study contributes and expands the knowledge about investor sentiment during crisis situations caused by external shocks.

## Literature review and research questions

### Investor sentiment and capital markets

The literature on behavioral finance has extensively explored whether investor sentiment affects trading decisions, leads to irrational trading behavior, and affects stock prices (e.g., Baker and Wurgler 2006; Beer and Zouaoui 2012; Chau et al. 2016; Cormier et al. 2010; Fang and Peress 2009; He et al. 2020; Hong and Stein 2007, 1999; Kaniel et al. 2008; Long et al. 1990; Palomino 1996; Shleifer and Vishny 1997). Bollen et al. (2011) find that investors’ trading behavior is directly shaped by their perceptions about future market development. Anxiety increases investors’ risk avoidance and contributes to pessimism (Baker and Wurgler 2006; Cen and Liyan 2013).

One relevant driver of investor perceptions and expectations is news. Tetlock (2007) shows that news media pessimism predicts downward pressure on market prices, even leading to a revision of fundamentals. Subsequent studies find that bad news leads to more intense or even panic-driven, long-term stock market effects (Cohen et al. 2018; Jung et al. 2018; Miller and Skinner 2015). A subordinate literature stream covers the formation and consequences of investor overreactions. In a fundamental study, de Bondt and Thaler (1985) investigate whether, and following which rules, the stock market overreacts. They present experimental evidence that in probability revision problems, people show a tendency to overreact, i.e., they overweight recent information and underweight base rate data. Barberis et al. (1998) link investor overreactions to news. They analyze the impact of good and bad market-relevant news announcements as a moderator of investor overreaction and stock return developments. Results show that investors overreact to consistent patterns of good (bad) news with a correlation to the length of good (bad) news series. Michayluk and Neuhauser (2006) analyze the role of investor overreaction during the market decline following the 1997 Asian financial crisis. They conclude that overreaction is sensitive to news announcements and is traceable for 1 week after the announcement. On a different note, Gennaioli et al. (2015) argue that a single piece of bad news in a series of good news items will not lead to a change in the underlying beliefs of investors regarding certain stocks. Nevertheless, a continuous series of bad news results in investor overreaction because previously ignored bad news is remembered, 'leading to a sharp rise in the perceived probability of a crisis and a collapse of prices' (Gennaioli et al. 2015, p.312).

Another influencing factor on investor sentiment is trust. Georgarakos and Pasini (2011) find an association between trust in financial markets and investors’ trading behavior. Based on a portfolio model using survey data, they show that in countries where investors exhibit a high level of trust toward the capital market, the stockholding share is significantly higher than in low-trust countries. Lesmeister et al. (2018) analyze whether shareholders extend or reduce their monitoring activity (e.g., by shareholder votes) relative to the general trust that they experience. The study reveals that trust reduces the amount of shareholder monitoring activity. Moreover, when exposed to external shocks, such as terrorist attacks, investors’ trust decreases by an increase in announced fatalities.

### Investor sentiment during epidemics/pandemics

As financial crises caused by epidemics or pandemics are not without precedent, few studies have taken the occasion of previous health crises to expand the knowledge of their power to impact investor sentiment. Funck and Gutierrez (2018) examine the impact of Ebola headline news on media-highlighted stocks. They employ the *VIX-Investor Fear Gauge* (VIX), introduced by Whaley (2009), to proxy for investor pessimism during Ebola outbreak announcements. They reveal that stock prices tend to reverse themselves within one day after the announcement, supporting the traditional theory of investor overreaction. Avian influenza (bird flu), initially reported in China in March 2013, caused a loss in the agriculture sector by $6.5 billion due to changes in prices, consumer confidence and trade volumes. Jiang et al. (2017) explored the impact of daily avian influenza case announcements on stock prices. They find peaks in investors’ overreacting behavior within the initial outbreak announcements; that is, investors seem to act more reasonably in time when the shock of the first outbreak news has been overcome.

Ichev and Marinč (2018) focus on the 2014–2016 Ebola outbreak. They analyze the effect of mass media news announcements about Ebola outbreak events on firms’ stock returns. They find that investors act irrationally to the news on the Ebola outbreak and that Ebola outbreak events unequally affect investors’ sentiment about stock returns, depending on the distance of the outbreak event from the markets. Negative effects on financial markets are stronger for firms that operate in countries with a larger Ebola exposure. They conclude that a firm’s geographic proximity to the Ebola outbreak event increases the impact on its stock returns.

In a recent study, Engelhardt et al. (2020) find that COVID-19-induced stock market declines in 64 countries are mainly associated with greater news attention and less with rational expectations. Following this research, we expect the growth rates of COVID-19 cases and deaths in firm revenue-relevant countries to depress investor sentiment by provoking panic and pessimism, resulting in temporary market overreactions and leading to a decline in stock returns during the pandemic.

### Government responses to the COVID-19 pandemic and investor sentiment

With rising COVID-19 cases and deaths, several governments worldwide took action to mitigate the repercussions of the disease and introduced diverse sets of countermeasures, i.e., government responses. In addition to restrictive policies to contain the spread of the disease (e.g., travel restrictions, stay-at-home requirements, school closings, public transport closings), stimulus packages were implemented to mitigate the economic impact of the pandemic (e.g., income support, debt relief, fiscal measures). Moreover, proactive measures were set in motion to reduce infection rates (e.g., testing policies, vaccination policies, contract tracing) (Hale et al. 2021). Following the literature on investor sentiment, we argue that government responses to COVID-19 can reinforce or mitigate the negative impact of the COVID-19 pandemic on stock prices. Governments may reduce uncertainty among investors, regain investors’ trust and lead to a less severe decline, or even rise, of firms’ stock returns, depending on the aim and scope of the policy. Moreover, we expect investors to rate government responses differently depending on a firm’s exposure to COVID-19; this is how severely those firm-relevant countries are affected, which directly contribute to the firm’s revenues.

## Data and research design

### COVID-19 data

We obtain daily data on worldwide confirmed COVID-19 cases and deaths per country from January 1, 2020 to March 15, 2021 from the *European Centre for Disease Prevention and Control (ECDC)*. We calculate the daily growth rates of both cases and deaths in each country, relative to a country’s population, by dividing the number of daily cases and deaths per million in country *i* on day *t* by the same measure of the previous day *t−*1. We receive a subsample of 180 countries and 563 observation days per country. Our sample is unbalanced since, for some sample countries, no cases or deaths were reported until April 2020.

### Capital market data

To measure firm-level stock market reactions to the COVID-19 pandemic, we obtain daily stock prices for 511 firms that make up the S&P 500 index during our observation period from January 1, 2020 to March 15, 2021. To solely assign COVID-19 cases and deaths and government responses of firm-relevant countries to a firm, we obtain country-specific sales revenues [REVENUE_i,c,t_] for the compounding S&P 500 firms for 2019 from the *FactSet Geographic Revenue (GeoRev)* Database. We assign all countries to a firm that contributed to the firm’s revenues, leading to a combination of multiple countries for each firm per observation day. We only include values with a certainty score of 70 or above, as provided by *GeoRev*. The certainty score is based on source metadata and ranges from 1 (low certainty) to 80 (declared value). This proceeding enables us to isolate information that is assumed to be of decision-relevant significance to each firm’s investors. See Appendix 1 for a list of countries included and the number of firms that realize revenues in the respective country.

### Government response data

We employ data from the OxCGRT, published by Hale et al. (2021), to measure country-specific government responses to the pandemic. Our dataset covers 16 individual ordinal scaled indicators that represent the strictness of various government policies in response to the COVID-19 pandemic. It should be noted that these values do not reflect the effectiveness of each policy. All indicators can be classified into three groups, representing the scope of the policy, i.e., containment and closure policies, health system policies, and economic policies. We include the following indicators:

Containment and closure policies

School closings; workplace closings; cancelations of public events; restrictions on gatherings; closing of public transport; stay-at-home requirements; restrictions on international movement; international travel controls

Health system policies

Public information campaigns; testing policy; contact tracing; facial covering policy; vaccination policy; protection of elderly people

Economic policies

Income support; debt and contract relief

Please see Appendix 2 for a full list of indicators, including detailed descriptions and scale coding. To reflect the extent of each government’s efforts, we calculate an index for each of the three policy groups.^1^ Since all indicators are ordinally scaled and ranked with different values set as maximums, we calculate subindices to normalize each indicator to an equally spaced scale between 0 and 100. The three indices, i.e., containment and closure index, health system index, and economic support index, are then calculated as simple averages of the normalized individual subindices. Moreover, we aggregate all three indices to create a summarized government response index. We merge our government response data with the firm-country dataset, including growth rates in the number of cases and deaths. Our final sample consists of 10,060,911 daily firm-country-specific observations.

### Empirical model

To test whether government responses to the COVID-19 pandemic moderate the impact of confirmed and announced COVID-19 cases and deaths on firms’ daily stock returns, we estimate the following regression model using firm- (Firm FE) and day-fixed (Day FE) effects*:

1 $$\begin{aligned} {\text{AR}}_{{{\text{i}},{\text{t}} + {1}}} & = \beta_{0} + \beta_{{1}} *{\text{ COVID}} - {19}_{{{\text{c}},{\text{t}}}} + \beta_{{2}} {\text{*RESPONSE}}_{{{\text{c}},{\text{t}}}} + \beta_{{3}} *{\text{COVID}} - {19}_{{{\text{c}},{\text{t}}}} \times {\text{ RESPONSE}}_{{{\text{c}},{\text{t}}}} \\ & \quad + \, \Sigma {\text{ Controls}}_{{{\text{i}},{\text{c}},{\text{t}}}} + {\text{ Firm FE}}_{{\text{i}}} + {\text{ Day FE}}_{{\text{t}}} + \, \varepsilon_{{{\text{i}};{\text{t}}}} \\ \end{aligned}$$

where *i*, *c*, and *t* index firm, country, and day, respectively.

Our dependent variable [AR_i,t+1_] is measured as adjusted abnormal logarithmic returns using a single-index market model, where expected returns are estimated with market betas using the firm’s daily stock returns and the S&P 500 index returns of the respective period (e.g., Brown and Barry 1984; Dai and Zhu 2020; Jain 1986; Sharpe 1963). Specifically, we define abnormal returns as $AR_{t}^{i} = R_{t}^{i} - E\left( {R_{t}^{i} } \right)$, where $R_{t}^{i}$ represents the daily return for firm *i* on day *t*. We estimate the firm’s expected return as $E(R_{t}^{i} ) = \alpha_{i} + b_{i} E(R_{t}^{market} )$, with $R_{t}^{market} = (P_{t}^{market} - P_{t - 1}^{market} )/P_{t - 1}^{market}$, where $P_{t}^{market}$ represents the S&P 500 index closing price on day *t*.^2^ Following prior research on the stock market impact of infectious diseases, our model parameters are estimated over a 90-trading-day estimation period, starting one day prior to day *t* to prevent unusual effects on the measurement day from interfering the estimation (e.g., Liu et al. 2020; Wang et al. 2013).

COVID-19_c,t_ represents either the growth rate of the announced cumulative number of confirmed COVID-19-positive cases [CASES_c,t_] or deaths [DEATHS_c,t_] associated with or caused by the disease per million in country *c* for day *t*. RESPONSE_c,t_ corresponds to each of our four daily government response indices, i.e., containment and closure index [CONTAINMENT_CLOSURE_c,t_], health system index [HEALTH_SYSTEM_c,t_], economic support index [ECON_SUPPORT_c,t_], and the summarized government response index [GOV_RESPONSE_c,t_]. We use a set of control variables based on prior literature. We control for abnormal returns one day prior to the measurement day by using abnormal returns [AR_*i,t*_] in autoregression to consider stock return autocorrelation (e.g., Kraft and Kraft 1977; Smirlock and Starks 1988). Zaremba et al. (2020) and Ali et al. (2020) reveal a significant impact of the COVID-19 pandemic on market volatility. Hence, we include a firm’s annualized volatility of logarithmic stock returns during our observation period [VOLATILITY_i,t_]. We control for the number of analysts following a firm’s share [FOLLOWING_i,t_] as well as for the percentage of institutional holdings [INSTITUTIONAL_i,t_], with both variables to be measured annually (e.g., Bartov et al. 2018; Chen et al. 2013; Hong et al. 2000). A broad number of studies investigate the relationship between firm fundamentals and stock market returns. We follow these findings by including firm size as proxied annually by the logarithm of a firm’s total assets [SIZE_i,t_] and the price-to-book ratio as a firm’s daily market price per share divided by the share’s book value [PTB_i,t_] (e.g., Fama and Franch 1993; Griffin and Lemmon 2002; Pontiff and Schall 1998). Moreover, corporate governance research finds that the number of a firm’s board members shapes board integrity and effectiveness and, thus, reinforced by the perception of investors, affects returns (Cheng 2008; González et al. 2019). Hence, we include the quarterly board size [BOARD_i,t_] in our set of controls. Davison (2020, p. 2) finds that 'the stocks returns of firms who are relatively unable to transition their business to comply with social distancing are much more responsive to changes in their level of leverage going into the pandemic'. We, therefore, process a firm’s leverage change during our observation period as the yearly differences in the ratio of a firm’s book value debt and total assets [LEVERAGE*i,t*]. Hu and Zhang (2021) show that firm performance deteriorates during the COVID-19 pandemic. However, the effect is smaller for firms in countries with better health systems, more advanced financial systems, and better institutions. We address these findings by controlling for the quarterly change in a firm’s return-on-assets [ROA_*i,t*_], calculated as operating income before depreciation over total assets. Several studies discuss a firm’s economic, social, and governmental scores (ESG scores) to indicate the resilience of stock prices during the COVID-19 pandemic. However, the results of preliminary studies are controversial. Albuquerque et al. (2020) find that stocks with higher ESG scores have significantly higher returns, lower return volatility, and higher operating profit margins during the first quarter of 2020. Broadstock et al. (2021) add that ESG performance mitigates financial risk during a financial crisis and that high-ESG portfolios generally outperform low-ESG portfolios. In contrast, Demers et al. (2020) conclude that higher ESG scores do not immunize stocks. We control for ESG scores [ESG_i,t_] using the most prevalent *Refinitiv* ESG *SCORE*^3^ that weekly measures a company’s relative ESG performance, commitment and effectiveness across 10 main themes, including emissions, environmental product innovation and human rights (Refinitiv 2020). Since the discussed literature on investor sentiment assigns an important role in affecting investor sentiment to news media, we control for three variables covering the spread, perception, and sentiment of COVID-19 news in each country. Data were obtained from the *RavenPack Coronavirus Media Monitor*. MEDIA_COVERAGEc*,t* calculates the daily percentage of all news agencies in country c on day t covering the topic of COVID-19. The index is computed as the daily number of distinct news agencies that mention COVID-19, divided by the total available number of news agencies in the country. MEDIA_HYPE_c,t_ measures the percentage of news that is currently reporting about COVID-19 in country c on day t, regardless the originating news agency. The index is computed as the daily number of reports that mention COVID-19, divided by the total daily number of reports. MEDIA_SENTIMENT_c,t_ measures the level of sentiment that news reports express towards a firm that is mentioned in the report alongside COVID-19. Specifically, it reflects the daily average of the difference between the number of positive and negative news reports. *RavenPack* determines positive or negative sentiment by 'systematically matching stories usually categorized by financial experts as having a positive or negative financial or economic impact. The algorithm produces a score for more than 6900 categories of business, economic, and geopolitical events, ranging from earnings announcements to natural disasters.' (Hafez et al. 2020). The index ranges between − 100 and 100, where a value of 100 is the most positive sentiment, − 100 is the most negative, and 0 is neutral. All variables are defined in Appendix 3.

## Empirical results

### Summary statistics

Table 1 reports descriptive statistics for all variables in our main regression model specifications. All variables are winsorized at the 1st and 99th percentiles to mitigate the influence of outliers. The mean value of firm-specific abnormal returns is 0.001 [AR_i,t+1_]. The mean annualized volatility of stock returns is 31.336 [VOLATILITY_i,t_], which is remarkable since the S&P 500’s annualized average volatility from 1926 through 2017 is 15.2.^4^ The mean share of revenues that a firm derives from a country is 1.126% [REVENUE_i,c,t_], with a maximum share of 53.175%. This maximum coincides with the average revenue share that S&P 500 firms realize in the United States, as expected. As the median value is 0.083 and the 75th percentile shows a value of 0.301, the highest revenue firms are allocated to a small number of countries. On average, a firm is followed by 22 analysts [FOLLOWING_i,t_], and the mean share of institutional owners [INSTITUTIONAL_i,t_] is 82.829%. Firm size [SIZE_i,t_], price-to-book-ratio [PTB_i,t_], and the level of leverage [LEVERAGE_i,t_] show mean values of 223.730 bn, 5.730, and − 0.973, respectively. A firm’s board comprises approximately 11 members. Unsurprisingly, concerning firm performance, the average return on assets is negative at – 1.656%. *Thomson Reuters Refinitiv* calculates a mean ESG score [ESG_i,t_] of 63.582 for our sample firms. Turning to country-specific data, on average, a country is exposed to a daily growth of 5.926% in COVID-19 cases per million people [CASES_i,t_] and a daily growth in COVID-19-related deaths per million people of 0.129% [DEATHS_c,t_]. We calculate our index for a country’s containment and closure policies [CONTAINMENT_CLOSURE_c,t_] to average 49.616. Values for our index covering a country’s efforts to support the health system [HEALTH_SYSTEM_c,t_] as well as the economy [ECON_SUPPORT_c,t_] rank closely at 54.449 and 43.965, respectively. Our summarized measure for a government’s effort in all fields [GOV_RESPONSE_c,t_] exhibits a mean value of 54.940. Our controls for the spread, perception, and sentiment of COVID-19 news in each country reveal the following descriptive insights: On average, 55.511% of all news agencies in a country cover the topic of COVID-19. The percentage of news that reports on COVID-19 each day in a country is 46.118. The mean level of sentiment toward a country mentioned in the news alongside COVID-19 is − 5.930, with an overall sample maximum of 50.950 of 100 index points and a minimum of − 97.210. The median remains negative at − 2.460.

**Table 1:** Descriptive statistics

Variable	Mean	Std	Min	25%	Median	75%	Max	N
Firm characteristics								
AR	0.001	2.107	− 6.606	− 1.056	− 0.021	1.019	7.190	155,855
VOLATILITY	31.336	10.380	15.948	24.014	29.405	35.321	66.822	155,855
FOLLOWING	21.604	8.008	5.000	16.000	21.000	26.000	46.000	155,855
INSTITUTIONAL	82.829	12.392	49.054	75.374	84.711	92.795	99.690	155,855
SIZE	232.730	370.861	12.651	47.178	110.660	236.010	2,455.100	155,855
PTB	5.730	32.942	− 194.226	1.671	3.858	7.752	176.193	155,855
BOARD	10.974	2.027	6.000	10.000	11.000	12.000	17.000	155,855
LEVERAGE	− 0.973	6.118	− 23.400	− 3.000	0.000	2.230	14.370	155,855
ROA	− 1.656	4.203	− 19.450	− 2.630	− 0.850	0.300	7.420	155,855
ESG	63.582	14.877	22.824	53.065	66.259	74.310	89.299	155,855
REVENUE	1.126	6.035	0.010	0.027	0.083	0.301	53.175	155,855
Country characteristics								
CASES	5.926	31.701	− 75.000	0.336	1.073	3.185	2200.265	54,900
DEATHS	0.129	1.458	− 3.704	0.000	0.112	0.045	113.953	54,900
CONTAINMENT_CLOSURE	49.616	27.904	0.000	31.250	54.688	71.875	100.000	54,900
HEALTH_SYSTEM	54.449	23.873	0.000	44.643	59.524	71.429	100.000	54,900
ECON_SUPPORT	43.965	33.230	0.000	0.000	50.000	75.000	100.000	54,900
GOV_RESPONSE	54.940	25.567	0.000	44.058	62.609	73.333	100.000	54,900
MEDIA_COVERAGE	55.511	21.086	0.060	43.270	59.500	71.250	100.000	54,900
MEDIA_HYPE	46.188	20.551	0.000	33.550	46.910	60.310	100.000	54,900
MEDIA_SENTIMENT	− 5.930	12.767	− 97.210	− 10.060	− 2.460	0.440	50.950	54,900
EXPOSURE	16.098	21.669	0.000	0.000	5.003	26.849	100.000	54,900
Observations in the firm-country-day-Matrix: 10,060,911								

This table provides the descriptive statistics for all variables. All variables are winsorized at the 1st and 99th percentiles

**Fig. 3:**
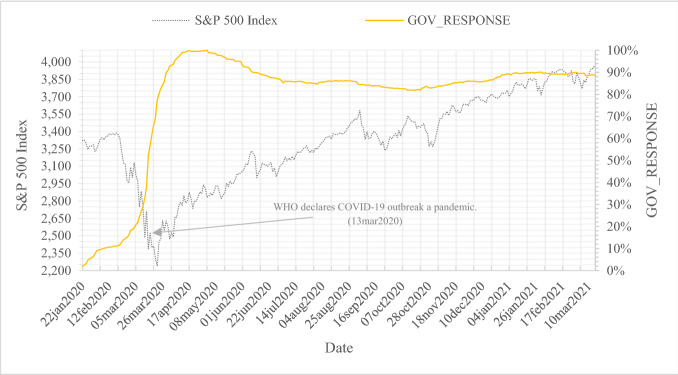
S&P 500 Index and global government response index. This figure shows the S&P 500 stock market index and the intensity of accumulated global government responses to COVID-19 for our observation period from January 1, 2020 to March 15, 2021. S&P 500 index data are derived from *Refinitiv Datastream*. Data for the calculation of the global government response intensity are provided by the *Oxford COVID-19 Government Response Tracker (OxCGRT)* database. The intensity of global government responses is calculated by accumulating all 16 policy indicators per day over all 180 countries. The scale is normalized on a range of 0 to 100, where 100 represents the maximum intensity for the observation period

Figure 3 shows the S&P 500 stock market index and the intensity of the global government response index [GOV_RESPONSE_c,t_] to COVID-19 for our observation period from January 1, 2020 to March 15, 2021. The intensity of global government responses is calculated by accumulating all 16 policy indicators per day over all 180 countries. The scale is normalized on a range of 0–100, where 100 represents the maximum intensity for the observation period. From March 5, 2020 on, worldwide initiatives to contain the spread of the pandemic are increasingly put in place. Hence, the global government response index grows exponentially. The growth accelerates from March 13, 2020, when the WHO declares the COVID-19 outbreak to officially be a pandemic. At the same time, the S&P 500 stock market index experiences a decrease that seemingly mirrors the government response’ s development. After a peak on April 17, 2020, the response index values diminish throughout the summer months, starting from approximately the date when the S&P 500 index recovers to its pandemic pre-announcement value. Towards winter, global government responses increase again slightly despite a continuously bullish stock market.

**Fig. 4:**
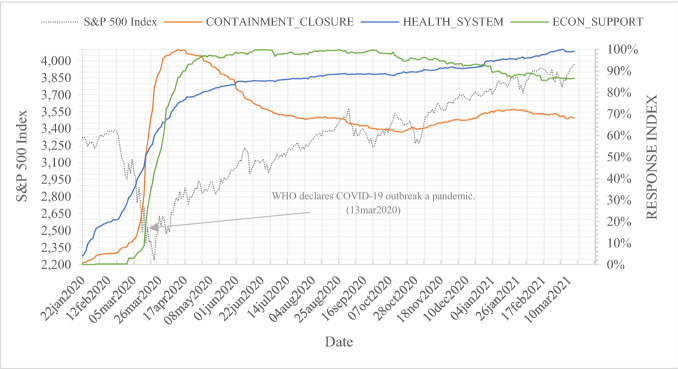
S&P 500 Index and global government responses by scope: containment and closure index, health system index and economic support index. This figure shows the S&P 500 stock market index and the intensity of global government responses in the fields of containment and closure, health systems, and economic support for COVID-19 for our observation period from January 1, 2020 to March 15, 2021. S&P 500 index data are derived from Thomson Reuters Datastream. Data for the calculation of the global government response intensity are provided by the Oxford COVID-19 Government Response Tracker (OxCGRT) database. The intensity of each global government response field is calculated by accumulating all field-specific policy indicators per day over all 180 countries. The scale is normalized on a range of 0 to 100, where 100 represents the maximum intensity for the observation period

Figure 4 shows the S&P 500 stock market index and the intensity of accumulated global government responses to COVID-19 for our observation period, split for policies in the fields of containment and closure [CONTAINMENT_CLOSURE_c,t_], health system [HEALTH_SYSTEM_c,t_], and economic support [ECON_SUPPORT_c,t_]. As before, the scale is normalized on a range of 0–100, where 100 represents the maximum intensity for the observation period. All three indices follow the path of the accumulated, global government response index, as illustrated in Fig. 3. However, health system initiatives are implemented later compared to containment and closure policies or economic support. Containment and closure policies experience the strongest decline, with health system initiatives still increasing. In the course of the bullish stock market, economic support and containment and closure policies are reduced, while health system support is still being extended.

Table 2 presents the Pearson–Spearman correlations. Both CASES_c,t_ and DEATHS_c,t_ are significantly negatively correlated with AR_i,t+1_ (Pearson − 0.042; Spearman − 0.029 and Pearson − 0.027; Spearman – 0.022). This is a first indicator of the negative impact that rising COVID-19 cases and deaths cause on stock prices. GOV_RESPONSE_c,t_ shows a significant and positive correlation with AR (Pearson 0.023; Spearman 0.019), as CONTAINMENT_CLOSURE_c,t_ does (Pearson 0.064; Spearman 0.055). In contrast, correlation coefficients for the remaining two indices, HEALTH_SYSTEM_c,t_ and ECON_SUPPORT_c,t_, are significantly negative regarding AR_i,t+1_ (Pearson − 0.032; Spearman − 0.041 and Pearson − 0.029; Spearman − 0.018). These findings imply that a split investigation of government responses is imperative since different government policies may provoke different market reactions. All four government response indices are significantly and positively correlated to both CASES_c,t_ and DEATHS_c,t_, exhibiting high magnitudes (e.g., COV_RESPONSE_c,t_ is correlated to CASES_c,t_ with Pearson 0.0552; Spearman 0.427). This illustrates the sensitivity of government interventions worldwide to rising COVID-19 cases and deaths. Turning to our controls, we find interesting values for our news media measures. As expected, both MEDIA_COVERAGE_c,t_ and MEDIA_HYPE_c,t_ are positively correlated with CASES_c,t_ and DEATHS_c,t_ (e.g., MEDIA COVEERAGE_c,t_ and CASES_c,t_ Pearson 0.192; Spearman 0.140). However, MEDIA_SENTIMENT_c,t_ is negatively correlated with COVID-19 proxies (CASES_c,t_: Pearson − 0.078; Spearman − 0.110, DEATHS_c,t_ Pearson − 0.124; Spearman − 0.123). This leads to the interpretation that the amount of news that mentions COVID-19 and specific countries increases as COVID-19 cases and deaths grow. At the same time, the sentiment expressed in the news media toward countries becomes negative as COVID-19 numbers grow. Overall, the absence of high correlations among our variables suggests that there are no multicollinearity concerns.

**Table 2:** Pearson–Spearman correlations

VARIABLE	TOTAL_ RETURN	AR	VOLA-TILITY	FOLLOW ING	INSTI-TUTIO-NAL	SIZE	PTB	BOARD	LEVE-RAGE	ROA	ESG	REVE-NUE	CASES	DEATHS	CONAIN-MENT_	HEALTH_ SYS-TEM	ECON_ SUP-PORT	GOV_ RES-PONSE	MEDIA_ COVER-AGE	MEDIA_ HYPE	MEDIA_ SENTI-MENT
															CLOSURE						
AR	0.594*		0.164*	0.018*	0.002*	− 0.056*	− 0.037*	− 0.031*	− 0.051*	− 0.009*	− 0.038*	− 0.006*	− 0.029*	− 0.022*	0.055*	− 0.041*	− 0.018*	0.019*	0.099*	0.091*	− 0.037*
VOLATILITY	0.222*	0.244*		0.114*	0.044*	− 0.175*	− 0.223*	− 0.080*	− 0.255*	− 0.204*	− 0.162*	− 0.027*	0.022*	0.021*	0.016*	0.018*	0.018*	0.020*	0.012*	0.008*	− 0.008*
FOLLOWING	0.018*	0.033*	0.146*		− 0.341*	0.431*	0.100*	0.087*	− 0.112*	− 0.070*	0.123*	− 0.004*	− 0.009*	− 0.009*	0.001*	− 0.004*	− 0.005*	− 0.002*	0.000	0.004*	0.002*
INSTITUTIONAL	− 0.031*	− 0.034*	− 0.099*	− 0.284*		− 0.521*	0.007*	− 0.251*	0.107*	0.059*	− 0.339*	0.006*	0.006*	0.006*	− 0.003*	0.001*	0.006*	0.000	0.002*	0.000	− 0.006*
SIZE	− 0.022*	− 0.030*	− 0.089*	0.366*	− 0.460*		− 0.098*	0.485*	0.009*	0.051*	0.471*	− 0.023*	− 0.003*	− 0.002*	0.000*	0.000	− 0.004*	− 0.001*	− 0.004*	− 0.003*	0.003*
PTB	− 0.013*	− 0.019*	− 0.039*	0.042*	− 0.013*	0.009*		− 0.109*	0.111*	0.173*	− 0.049*	0.014*	0.023*	0.016*	− 0.034*	0.022*	− 0.006*	− 0.017*	− 0.055*	− 0.048*	0.016*
BOARD	− 0.011*	− 0.029*	− 0.067*	0.066*	− 0.199*	0.172*	− 0.014*		− 0.048*	− 0.051*	0.297*	− 0.005*	0.001*	0.000	− 0.001*	0.003*	0.001	0.000*	− 0.004*	− 0.004*	0.002*
LEVERAGE	− 0.096*	− 0.105*	− 0.382*	− 0.084*	0.201*	0.015*	0.039*	− 0.086*		0.287*	− 0.052*	− 0.015*	0.003*	0.005*	0.000	− 0.001*	0.002*	0.001*	0.002*	0.000	− 0.007*
ROA	− 0.105*	− 0.094*	− 0.387*	− 0.066*	0.197*	0.099*	0.062*	− 0.066*	0.473*		0.041*	− 0.007*	− 0.009*	− 0.007*	− 0.007*	− 0.012*	− 0.009*	− 0.009*	0.000	− 0.001*	− 0.003*
ESG	− 0.024*	− 0.035*	− 0.124*	0.093*	− 0.295*	0.317*	0.007*	0.261*	− 0.026*	0.072*		0.011*	− 0.018*	− 0.019*	− 0.004*	− 0.011*	− 0.017*	− 0.011*	− 0.009*	− 0.003*	0.014*
REVENUE	0.002*	0.002*	0.002*	− 0.016*	0.005*	0.001*	− 0.004*	0.001*	0.002*	0.000	− 0.023*		0.175*	0.228*	0.076*	0.205*	0.194*	0.177*	0.175*	0.028*	− 0.278*
CASES	0.043*	-0.042*	0.023*	− 0.011*	0.004*	− 0.003*	0.038*	0.000	− 0.002*	− 0.017*	− 0.018*	0.105*		0.832*	0.409*	0.555*	0.427*	0.533*	0.173*	0.140*	− 0.110*
DEATHS	0.035*	− 0.027*	0.019*	− 0.010*	0.005*	− 0.001	0.031*	0.000	− 0.002*	− 0.013*	− 0.018*	0.148*	0.779*		0.427*	0.435*	0.348*	0.506*	0.184*	0.137*	− 0.123*
CONTAINMENT_ CLOSURE	0.122*	0.064*	0.020*	0.001*	− 0.005*	0.002*	0.000	− 0.001*	− 0.003*	− 0.012*	− 0.005*	0.037*	0.426*	0.349*		0.380*	0.321*	0.888*	0.446*	0.431*	− 0.122*
HEALTH_SYSTEM	0.064*	− 0.032*	0.022*	− 0.005*	− 0.001*	− 0.001	0.035*	0.001*	− 0.004*	− 0.018*	− 0.010*	0.059*	0.562*	0.370*	0.568*		0.525*	0.679*	0.194*	0.157*	− 0.104*
ECON_SUPPORT	0.057*	− 0.029*	0.019*	− 0.006*	0.005*	− 0.003*	0.009*	− 0.001*	− 0.002*	− 0.016*	− 0.017*	0.037*	0.429*	0.308*	0.410*	0.602*		0.594*	0.224*	0.212*	− 0.117*
GOV_RESPONSE	0.111*	0.023*	0.024*	− 0.002*	− 0.003*	0.001	0.014*	− 0.001*	− 0.004*	− 0.017*	− 0.010*	0.051*	0.552*	0.411*	0.911*	0.832*	0.667*		0.388*	0.354*	− 0.157*
MEDIA_COVERAGE	0.104*	0.121*	0.014*	0.001*	0.000	0.003*	− 0.017*	− 0.004*	0.001	− 0.007*	− 0.009*	0.106*	0.192*	0.145*	0.518*	0.386*	0.297*	0.521*		0.885*	− 0.206*
MEDIA_HYPE	0.101*	0.119*	0.009*	0.005*	− 0.001*	0.000	− 0.019*	− 0.005*	− 0.001*	− 0.004*	− 0.002*	− 0.056*	0.148*	0.102*	0.493*	0.325*	0.255*	0.476*	0.902*		− 0.174*
MEDIA_SENTIMENT	− 0.021*	− 0.058*	− 0.008*	0.002*	− 0.005*	− 0.004*	0.009*	0.002*	− 0.003*	0.003*	0.012*	− 0.031*	− 0.078*	− 0.124*	− 0.130*	− 0.106*	− 0.083*	− 0.136*	− 0.213*	− 0.168*	

This table presents Pearson–Spearman correlations. Measures above and below the diagonal represent Spearman and Pearson correlations, respectively. Values with * indicator mark coefficients that are significant at p < 0.05. All variables are winsorized at the 1st and 99th percentiles

### Government responses and stock returns

First, we examine whether the impact of COVID-19 on firms’ daily abnormal stock returns is moderated by the summarized government responses of countries that contribute to a firm’s revenue. Table 3 presents our main regression results for Eq. (1).

**Table 3:** Government responses and stock returns

Dependent variable	(I)	(II)	(III)	(IV)
	Abnormal return [AR_t+1_]			
CASES	− 0.038*** (− 125.203)		− 0.043*** (− 40.616)	
DEATHS		− 0.063*** (− 81.551)		− 0.184*** (− 13.942)
GOV_RESPONSE			− 0.000***	− 0.025***
			(− 2.912)	(− 33.890)
CASES × GOV_RESPONSE			0.000***	
			(5.493)	
DEATHS × GOV_RESPONSE				0.031*** (9.905)
VOLATILITY	0.017*** (5.910)	0.018*** (6.088)	0.017*** (5.919)	0.018*** (6.033)
AR	0.037*** (72.478)	0.039*** (77.118)	0.037*** (72.460)	0.039*** (76.430)
FOLLOWING	0.010*** (3.763)	0.010*** (3.677)	0.010*** (3.762)	0.010*** (3.682)
INSTITUTIONAL	0.004 (1.637)	0.003 (1.471)	0.004 (1.632)	0.003 (1.507)
SIZE	0.000 (0.440)	0.000 (0.339)	0.000 (0.436)	0.000 (0.367)
PTB	− 0.001*** (− 30.429)	− 0.001*** (− 34.522)	− 0.001*** (− 30.566)	− 0.001*** (− 33.306)
BOARD	0.058*** (4.825)	0.056*** (4.674)	0.058*** (4.819)	0.056*** (4.712)
LEVERAGE	0.007*** (4.489)	0.006*** (4.442)	0.007*** (4.484)	0.007*** (4.461)
ROA	− 0.010 (− 1.082)	-0.008 (− 0.851)	− 0.010 (− 1.072)	− 0.009 (− 0.919)
ESG	− 0.006*** (− 5.258)	− 0.006*** (− 5.083)	− 0.006*** (− 5.251)	− 0.006*** (− 5.131)
REVENUE	− 0.000 (− 0.080)	− 0.000 (− 0.033)	− 0.000 (− 0.082)	− 0.000 (− 0.031)
MEDIA_COVERAGE	0.003*** (38.495)	0.003*** (33.332)	0.003*** (35.795)	0.003*** (40.035)
MEDIA_HYPE	0.001*** (14.073)	0.001*** (12.261)	0.001*** (14.047)	0.001*** (12.826)
MEDIA_SENTIMENT	− 0.003*** (− 54.836)	− 0.003*** (− 59.805)	− 0.003*** (− 55.117)	− 0.003*** (− 57.303)
Constant	− 1.066*** (− 3.316)	− 1.126*** (− 3.500)	− 1.078*** (− 3.355)	− 1.067*** (− 3.316)
Firm FE	Yes	Yes	Yes	Yes
Day FE	Yes	Yes	Yes	Yes
Observations	3,829,441	3,829,441	3,829,441	3,829,441
Adj. R^2^	0.0410	0.0388	0.0410	0.0391
F-test (CASES/DEATHS = GOV_RESPONSE)			< 0.01	< 0.01

This table reports the regression results for the association between the daily growth in COVID-19 case [CASES] and death [DEATH] numbers and a firm’s abnormal returns [AR] both solely and depending on summarized government responses to the COVID-19 pandemic [GOV_RESPONSE]. See Appendix 3 for all variable definitions. Models I and II report the results for the impact of daily growth in cases and deaths, respectively. Models III and IV report the interaction with summarized government responses for the growth in the number of both cases and deaths. All continuous variables are winsorized to the 1st and 99th percentiles of their distributions. The t-statistics from robust standard errors clustered at firm level are presented in parentheses

*, **, ***Significance at 10, 5, and 1% based on two-tailed tests

Models I and II examine the raw impact of the growth rate of the announced number of confirmed COVID-19-positive cases [CASES_c,t_] or deaths [DEATHS_c,t_] per million associated with or caused by the disease on abnormal returns [AR_i,t+1_]. As expected, results show that the stock market responds significantly negatively to both COVID-19 proxies. However, the daily growth in the number of country-specific deaths is of greater relevance to investors since the coefficient for DEATHS_c,t_ (− 0.063) is larger in magnitude than the coefficient for CASES_c,t_ (− 0.038). These findings are contrary to previous studies on the impact of COVID-19 cases and deaths on the stock market that expose cases to mainly drive the stock market (e.g., Alexakis et al. 2021; Ali et al. 2020; Erdem 2020). This contrast is most likely explained by the longer observation period that is considered in our study. Specially, when analyzing the consequences of diseases for the first time, a longer observation period allows for incorporating the different stages it passes. More specifically, in the initial period, the spread of the pandemic was mainly measured (and publicly discussed) by the increasing number of cases. In the course of the pandemic, this perspective changed due to worldwide high levels of cases, and deaths became a more important focus of interest for health organizations, governments, and news media. In addition, as is shown in Fig. 1, the increase in the number of deaths was delayed compared to the increase in the number of cases. Hence, due to short observations covering the initial period of the pandemic, most preliminary studies lack enough data to reveal robust and interpretable results concerning deaths.

Our firm specific controls perform as expected and support the findings of the previous studies on stock market reaction we discussed. Turning to our media-related controls, we find MEDIA_COVERAGE and MEDIA_HYPE to show positive effects on stock returns. This is unsurprising since prior research on behavioral finance has well explored that the excessive presence of news, regardless of the expressed sentiment, leads to a higher attention of investors, and thus, gives positive momentum to the stock market development (e.g., Andrei and Halser 2015; Engelhardt et al. 2020). At the same time, MEDIA_SENTIMENT shows a significant and negative impact on stock returns. As this variable incorporates both positive and negative sentiment, and descriptive statistics reveal a mean of − 5.930, and thus, predominantly negative sentiment throughout our observation period, this result follows prior research that finds negative news to negatively influence the stock markets (e.g., Cohen et al. 2018; Jung et al. 2018).

In Models III and IV, we add our summarized government response index [GOV_RESPONSE_c,t_] to analyze whether the entirety of a country’s responses to the pandemic moderates the association shown in the previous models. Across both models, we find positive and statistically significant coefficients on the moderation of cases and deaths by government responses. This indicates that the entirety of government policies positively influences investor sentiment, retriggers optimism, restores investor trust and eventually mitigates the decline of stock prices. In other words, market participants seem to appreciate governments’ efforts to contain the consequences of the pandemic. Our control variables follow similar patterns as in the previous models. Within each regression, F tests indicate that the coefficients on the CASES/DEATHS and GOV_RESPONSE variables are significantly different from each other in all specifications, suggesting that both variables add explanatory value to our model.

### Government responses split by scope and stock returns

Since we find summarized government responses to play an important role in stock market development during the COVID-19 pandemic, we are now interested in dividing our GOV_RESPONSE_c,t_ index into the different scopes of government responses, measured by our 16 country-specific indicators, to see whether different scopes of responses mitigate or reinforce the negative stock market impact of the pandemic. Table 4 presents the results of our regression models covering the moderating effects of government containment and closure policies [CONTAINMENT_CLOSURE_c,t_], the support of the country’s health system [HEALTH_SYSTEM_c,t_], and the support of the economy [ECON_SUPPORT_c,t_].

**Table 4:** Government responses by scope and stock returns

Dependent variable	(I)	(II)	(III)	(IV)	(V)	(VI)
	Abnormal return [AR_t+1_]					
CASES	− 0.073*** (− 37.388)		− 0.077*** (28.866)		− 0.033*** (− 42.144)	
DEATHS		− 0.277*** (− 33.576)		− 0.172*** (19.031)		− 0.114*** (− 45.422)
CONTAINMENT_CLOSURE	0.015* (21.586)c	− 0.012*** (− 18.660)				
HEALTH_SYSTEM			− 0.008*** (− 10.834)	− 0.035*** (− 49.668)		
ECON_SUPPORT					− 0.026*** (− 50.218)	− 0.039*** (− 93.019)
CASES × CONTAINMENT_CLOSURE	0.007*** (15.514)					
DEATHS × CONTAINMENT_CLOSURE		0.053*** (26.712)				
CASES × HEALTH_SYSTEM			− 0.034*** (− 43.061)			
DEATHS × HEALTH_SYSTEM				− 0.059*** (− 24.756)		
CASES × ECON_SUPPORT					0.001*** (5.315)	
DEATHS × ECON_SUPPORT						0.019*** (29.899)
VOLATILITY	0.017*** (5.866)	0.018*** (6.026)	0.017*** (5.933)	0.018*** (6.027)	0.017*** (5.915)	0.018*** (6.005)
AR	0.037*** (72.001)	0.039*** (76.540)	0.036*** (71.132)	0.038*** (75.180)	0.036*** (70.534)	0.037*** (72.156)
FOLLOWING	0.010*** (3.829)	0.010*** (3.710)	0.010*** (3.726)	0.010*** (3.669)	0.010*** (3.724)	0.009*** (3.665)
INSTITUTIONAL	0.004* (1.693)	0.003 (1.524)	0.003 (1.609)	0.003 (1.511)	0.004 (1.615)	0.003 (1.520)
SIZE	0.000 (0.468)	0.000 (0.374)	0.000 (0.333)	0.000 (0.311)	0.000 (0.409)	0.000 (0.357)
PTB	− 0.001*** (− 30.306)	− 0.001*** (− 33.608)	− 0.001*** (− 29.888)	− 0.001*** (− 32.371)	− 0.001*** (− 30.399)	− 0.001*** (− 32.756)
BOARD	0.058*** (4.883)	0.057*** (4.733)	0.057*** (4.816)	0.057*** (4.731)	0.057*** (4.779)	0.056*** (4.676)
LEVERAGE	0.007*** (4.505)	0.007*** (4.459)	0.007*** (4.561)	0.007*** (4.527)	0.007*** (4.462)	0.006*** (4.411)
ROA	− 0.011 (− 1.135)	− 0.009 (− 0.929)	− 0.010 (− 1.071)	− 0.009 (− 0.940)	− 0.010 (− 1.073)	− 0.009 (− 0.947)
ESG	− 0.006*** (− 5.307)	− 0.006*** (− 5.149)	− 0.006*** (− 5.254)	− 0.006*** (− 5.153)	− 0.006*** (− 5.242)	− 0.006*** (− 5.144)
REVENUE	− 0.000 (− 0.074)	− 0.000 (− 0.030)	− 0.000 (− 0.066)	− 0.000 (− 0.019)	− 0.000 (− 0.102)	− 0.000 (− 0.068)
MEDIA_COVERAGE	0.003*** (32.938)	0.003*** (36.599)	0.003*** (38.949)	0.003*** (42.572)	0.003*** (44.552)	0.003*** (43.849)
MEDIA_HYPE	0.001*** (12.513)	0.001*** (12.647)	0.001*** (12.930)	0.001*** (10.919)	0.001*** (15.852)	0.001*** (16.549)
MEDIA_SENTIMENT	− 0.003*** (− 54.363)	− 0.003*** (− 56.592)	− 0.003*** (− 51.490)	− 0.003*** (− 55.408)	− 0.002*** (− 47.135)	− 0.002*** (− 45.855)
Constant	− 1.086*** (− 3.381)	− 1.105*** (− 3.434)	− 1.049*** (− 3.265)	− 1.037*** (− 3.226)	− 1.044*** (− 3.249)	− 1.067*** (− 3.321)
Firm FE	Yes	Yes	Yes	Yes	Yes	Yes
Day FE	Yes	Yes	Yes	Yes	Yes	Yes
Observations	3,829,441	3,829,441	3,829,441	3,829,441	3,829,441	3,829,441
Adj. R^2^	0.0412	0.0390	0.0415	0.0396	0.0418	0.0409
F test CASES/DEATHS = CONTAINMENT_CLOSURE	< 0.01	< 0.01				
HEALTH_SYSTEM			< 0.01	< 0.01		
ECON_SUPPORT					< 0.01	< 0.01

This table reports the regression results for the association between the daily growth in COVID-19 case [CASES] and death [DEATH] numbers and a firm’s abnormal returns [AR] and the dependence of split government responses to the COVID-19 pandemic in the fields of containment and closure [CONTAINMENT_CLOSURE], health system policies [HEALTH_SYSTEM], and economic support [ECON_SUPPORT]. See Appendix 3 for all variable definitions. Models I and II report the results for the interaction of the daily growth in cases and deaths with containment and closure policies. Models III and IV report the interaction of the daily growth in cases and deaths with health system policies, and Models V and VI report the interaction of the daily growth in cases and deaths with government economic support. All continuous variables are winsorized to the 1st and 99th percentiles of their distributions. The t-statistics from robust standard errors clustered at firm level are presented in parentheses

*, **, ***Significance at 10, 5, and 1% based on two-tailed tests

In Models I and II, we estimate the additional moderating effect of containment and closure policies in coherence with CASES_c,t_ and DEATHS_c,t_, respectively. Both interaction coefficients are positive and statistically significant. Hence, governments mitigate negative market impacts of the pandemic by taking actions to contain the spread of the disease, e.g., school closings, workplace closings, closings of public transport or issuing stay-at-home orders.

Government economic support [ECON_SUPPORT_c,t_] in Models V and VI has similar effects. Investors seem to appreciate the efforts of governments to mitigate the economic consequences of the pandemic by relieving debts and contracts or by supporting incomes. They most likely adjust their perceptions about market development and, in consequence, positively adjust their investment decisions.

Interestingly, government support of a country’s health system [HEALTH_SYSTEM_c,t_], shown in Models III and IV, causes further declines in abnormal stock returns as the interaction coefficients are negative and significant. For the interpretation of this counterintuitive effect, it is helpful to reinvestigate Fig. 4. The pathway of the index for government efforts globally in supporting health systems [HEALTH_SYSTEM_c,t_] differs from the remaining two indices. From the date the WHO pronounced COVID-19 a pandemic on March 13, 2020 until the S&P 500 recovered to a preannouncement value on March 30, 2020, the growth of the health system index was significantly smaller, suggesting a smaller contribution to stock market declines. As the situation progressed, with recovering S&P 500 values, the health system index continued growing, while the other two indices showed persistent declines. Analyzing the index composition, one explanation may be the initial weakness and the delay of efforts in strengthening the health system. For example, widespread testing initiatives were implemented late due to the absence of reliable tests. This more obviously holds true for vaccination campaigns. Because health system policies are mainly implemented when the stock market was on a path of recovery and was gaining momentum, investors apparently feared restrictions or policies in the health sector that would interfere with the boom again. This uncertainty most likely provoked pessimism and, in consequence, negative stock market effects. Results from F tests indicate a significant difference in the coefficients on CASES/DEATHS and the government response measures.

The coefficients of our sets of firm and country-specific control variables remain stable in significance, magnitude, and direction with indistinguishable differences from our previous regression covering summarizes government responses.

### Government responses and firm-specific COVID-19 revenue exposure

In contrast to most studies on the economic consequences of the COVID-19 pandemic we discussed, we investigate COVID-19 related impacts on firm-level rather than aggregating an entire economy. This approach allows a detailed investigation of the linkage between the COVID-19 pandemic, worldwide government regulations, and multinational firms’ characteristics.

One major field of interest for both research and practice is whether some firms are more severely affected by the COVID-19 pandemic and by government responses than others. Recent studies show various firm-specific characteristics to moderate the extent of the declines in stock returns associated with the pandemic. For example, Ding et al. (2021) find that firms with high exposure to supply chain disruptions show greater stock price declines.

We aim to analyze whether country-specific sales revenues at the firm level influence the effect of governmental responses to COVID-19-associated stock market effects. Specifically, we are interested in whether investors react differently to a growth in the number of COVID-19 cases and deaths and related government responses when a firm is more severely affected by this growth on the sales side.

We employ the comprehensive *FactSet Geographic Revenue Exposure (GeoRev)* Database that meters a firm’s annual sales revenue for each of the world's countries it operates in. The data is derived from a broad set of sources, e.g., summarized annual reports. In addition, 'an estimation algorithm based on GDP weighting and accounting logic is then applied to solve for any non-explicit disclosures' (FactSet 2022). The result is a detailed breakdown of a company’s revenues into any geographic country. The database also provides a certainty score that is based on source metadata and ranges from 1 (low certainty) to 80 (declared value). We only include values with a certainty score of 70 or above.

We assume, that for each firm, country-specific revenues reflect the importance of a country to the firm’s economy. The risk of losing revenue in a country may increase when the COVID-19 pandemic’s intensity is high. To combine both the importance of a country for a firm’s revenues and the risk of losses in a country, we calculate an indicator for firm-country-level sales revenue exposure to the pandemic. Thus, EXPOSURE_i,t_ is the country-specific sales revenues of our sample firms [REVENUE_i,c,t_], weighted by country’s daily growth rates of COVID-19 cases [CASES_c,t_] and deaths [DEATHS_c,t_] per million. For instance, a high infection or death rate in a country that does not significantly contribute to a firm’s sales revenues may not be considered as a threat to that firm. The same may holds true for a country that highly contributes to the firm’s sales revenue but shows low infection rates.

The variable is then standardized using z-scores and normalized to a scale from 0 to 100, where higher values indicate a larger firm-specific revenue exposure to COVID-19. For example, a value of 100 would be related to a firm-day observation if the growth in COVID-19 cases or deaths among all firm-relevant countries is the highest in the countries that contribute most to the firm’s revenues. In the same way, a value of 0 would be related to a firm-day observation if there is no growth in COVID-19 cases or deaths among all countries that contribute to a firm’s revenues. We make the following adjustments to our regression model in Eq. (1):

2 $$\begin{gathered} {\text{AR}}_{{{\text{i}},{\text{t}} + {1}}} = \beta_{0} + \beta_{{1}} *{\text{ COVID}} - {19}_{{{\text{c}},{\text{t}}}} + \beta_{{2}} *{\text{RESPONSE}}_{{{\text{c}},{\text{t}}}} + \beta_{{3}} *{\text{EXPOSURE}}_{{{\text{i}},{\text{t}}}} + \hfill \\ \beta_{{4}} {\text{* COVID}} - {19}_{{{\text{c}},{\text{ t}}}} \times {\text{ RESPONSE}}_{{{\text{c}},{\text{t}}}} \times {\text{ EXPOSURE}}_{{{\text{i}},{\text{t}}}} + \, \Sigma {\text{ Controls}}_{{{\text{i}},{\text{c}},{\text{t}}}} + {\text{ Firm FE}}_{{\text{i}}} + \hfill \\ {\text{Day FE}}_{{\text{t}}} + \, \varepsilon_{{{\text{i}};{\text{t}}}} \hfill \\ \end{gathered}$$

where i, c, and t index firm, country, and day, respectively.

Results are presented in Table 5, where Panels A and B observe CASES_c,t_ and DEATHS_c,t,_ respectively, as proxies for COVID-19_c,t_.

**Table 5:** Government responses and firm-specific COVID-19 revenue exposure

Dependent variable	(I)	(II)	(III)	(IV)
	Abnormal return [AR_t+1_]			
A [CASES]				
CASES	− 0.041*** (− 28.423)	− 0.058*** (− 22.357)	− 0.041*** (9.393)	− 0.031*** (− 34.138)
EXPOSURE	0.041*** (7.636)	− 0.034*** (− 3.403)	0.808*** (45.111)	0.047*** (13.170)
CASES × EXPOSURE	− 0.004*** (− 3.469)	0.011*** (5.667)	− 0.138*** (− 38.297)	− 0.010*** (− 12.538)
GOV_RESPONSE	− 0.001*** (− 16.633)			
CONTAINMENT_CLOSURE		0.001* (1.670)		
HEALTH_SYSTEM			− 0.013*** (− 17.015)	
ECON_SUPPORT				− 0.026*** (− 52.973)
CASES × GOV_RESPONSE	0.000*** (14.821)			
CASES × CONTAINMENT_CLOSURE		0.009*** (13.367)		
CASES × HEALTH_SYSTEM			− 0.015*** (− 14.150)	
CASES × ECON_SUPPORT				0.005*** (19.937)
GOV_RESPONSE × EXPOSURE	− 0.001*** (− 9.987)			
CONTAINMENT_CLOSURE × EXPOSURE		0.005* (1.875)		
HEALTH_SYSTEM × EXPOSURE			− 0.196*** (− 46.008)	
ECON_SUPPORT × EXPOSURE				− 0.012*** (− 13.689)
CASES × GOV_RESPONSE × EXPOSURE	0.000*** (4.515)			
CASES × CONTAINMENT_CLOSURE × EXPOSURE		− 0.002*** (− 4.912)		
CASES × HEALTH_SYSTEM × EXPOSURE			0.033*** (39.226)	
CASES × ECON_SUPPORT × EXPOSURE				0.002*** (10.871)
CONTROLS	Yes	Yes	Yes	Yes
Constant	− 0.247*** (− 33.948)	− 0.257*** (− 35.102)	− 0.230*** (− 30.807)	− 0.238*** (− 32.872)
Firm FE	Yes	Yes	Yes	Yes
Day FE	Yes	Yes	Yes	Yes
Observations	3,829,441	3,829,441	3,829,441	3,829,441
Adj. R^2^	0.0256	0.0255	0.0266	0.0264
F test				
CASES = EXPOSURE	< 0.05	< 0.01	< 0.01	< 0.05
CASES = GOV_RESPONSE/CONTAINMENT_CLOSURE/HEALTH_SYSTEM/ECON_SUPPORT	< 0.01	< 0.01	< 0.01	< 0.01
EXPOSURE = GOV_RESPONSE/CONTAINMENT_CLOSURE/HEALTH_SYSTEM/ECON_SUPPORT	< 0.01	< 0.05	< 0.01	< 0.01
B **[DEATHS]**				
DEATHS	− 0.132*** (− 22.084)	− 0.233*** (− 17.480)	− 0.013*** (− 0.790)	− 0.108*** (− 30.839)
EXPOSURE	− 0.024*** (− 8.861)	− 0.060*** (− 11.902)	0.310*** (33.241)	0.001 (0.588)
DEATHS × EXPOSURE	0.028*** (12.764)	0.052*** (9.832)	− 0.063*** (− 8.934)	0.002 (1.269)
GOV_RESPONSE	− 0.001*** (− 32.539)			
CONTAINMENT_CLOSURE		− 0.008*** (− 12.873)		
HEALTH_SYSTEM			− 0.025*** (− 34.979)	
ECON_SUPPORT				− 0.027*** (− 66.425)
DEATHS × GOV_RESPONSE	0.001*** (17.291)			
DEATHS × CONTAINMENT_CLOSURE		0.047*** (14.631)		
DEATHS × HEALTH_SYSTEM			− 0.006 (− 1.496)	
DEATHS × ECON_SUPPORT				0.023*** (25.318)
GOV_RESPONSE × EXPOSURE	− 0.000 (− 0.262)			
CONTAINMENT_CLOSURE × EXPOSURE		0.008*** (6.173)		
HEALTH_SYSTEM × EXPOSURE			− 0.079*** (− 36.098)	
ECON_SUPPORT × EXPOSURE				− 0.005*** (− 8.881)
DEATHS × GOV_RESPONSE × EXPOSURE	0.000*** (− 9.930)			
DEATHS × CONTAINMENT_CLOSURE × EXPOSURE		− 0.011*** (− 8.380)		
DEATHS × HEALTH_SYSTEM × EXPOSURE			0.017*** (10.324)	
DEATHS × ECON_SUPPORT × EXPOSURE				0.000 (0.439)
CONTROLS	Yes	Yes	Yes	Yes
Constant	− 0.252*** (− 34.690)	− 0.259*** (− 35.463)	− 0.213*** (− 28.628)	− 0.247*** (− 34.084)
Firm FE	Yes	Yes	Yes	Yes
Day FE	Yes	Yes	Yes	Yes
Observations	3,829,441	3,829,441	3,829,441	3,829,441
Adj. R^2^	0.0251	0.0249	0.0258	0.0262
F test				
DEATHS = EXPOSURE	< 0.05	< 0.05	< 0.01	< 0.01
DEATHS = GOV_RESPONSE/CONTAINMENT_CLOSURE/HEALTH_SYSTEM/ECON_SUPPORT	< 0.01	< 0.01	< 0.01	< 0.01
EXPOSURE = GOV_RESPONSE/CONTAINMENT_CLOSURE/HEALTH_SYSTEM/ECON_SUPPORT	< 0.01	< 0.01	< 0.01	< 0.01

This table reports the regression results for the association between the daily growth in the number of COVID-19 cases [CASES] and deaths [DEATH] and a firm’s abnormal returns [AR] depending on (1) summarized government responses [GOV_RESPONSES] and split government responses to the COVID-19 pandemic in the fields of containment and closure [CONTAINMENT_CLOSURE], health system policies [HEALTH_SYSTEM], and economic support [ECON_SUPPORT] and (2) a firm’s specific daily revenue exposure to COVID-19 [EXPOSURE]. See Appendix 3 for all variable definitions. In Panel A, Model I presents the results for the interaction of the daily growth in COVID-19 case numbers with summarized government responses to the COVID-19 pandemic. Models II to IV report the interaction of the daily growth in COVID-19 case numbers with containment and closure policies (Model II), health system policies (Model III), and government economic support (Model IV). In Panel B, Model I presents the results for the interaction of the daily growth in COVID-19 death numbers with summarized government responses to the COVID-19 pandemic. Models II to IV report the interaction of the daily growth in COVID-19 death numbers with containment and closure policies (Model II), health system policies (Model III), and government economic support (Model IV). All continuous variables are winsorized to the 1st and 99th percentiles of their distributions. The t-statistics from robust standard errors clustered at the firm level are presented in parentheses

*, **, ***Significance at 10, 5, and 1% based on two-tailed tests

In Panel A Model I, the three-way interaction reveals that the mitigating effect of summarized government responses [GOV_RESPONSE_c,t_], as shown previously, is even stronger for firms that are highly exposed to COVID-19 on the sales side. The coefficient is positive and significant but shows a low magnitude.

The same holds true for governmental efforts in economic support [ECON_SUPPORT_c,t_], presented in Model IV. In contrast, containment and closure policies [CONTAINMENT_CLOSURE_c,t_] (Model II) seem to be rated differently by investors in the case that a firm’s revenue is highly exposed to COVID-19. While in our main regression model with split government responses by scope, containment and closure policies mitigate stock price declines due to growing COVID-19 case numbers, they seem not strong enough to do so if a firm achieves high sales revenues in countries that are highly affected by the pandemic.

Turning to health system policies [HEALTH_SYSTEMc,t] (Model III), government support seems to be rated more positively when investors are more severely affected by the pandemic. Moreover, the additional positive effect of government health system policies for firms that are highly exposed to the pandemic (coefficient 0.033) even reverses the initial negative effect (coefficient − 0.015), where the moderation of EXPOSURE_c,t_ was unconsidered. Thus, investors of firms with high COVID-19 revenue exposure aim for a fast recovery and positively assess health system policies, regardless of consequential restrictions. In Panel B, COVID-19_c,t_ is represented by DEATHS_c,t._ For all models, the results are similarly directed. As a sole exception, we fail to find a significant three-way interaction for the economic support index.

### Estimation of COVID-19 growth rates with an epidemiological standard model

In our main regression models, we employ the percentage growth rates for both COVID-19 cases and deaths to proxy for the spread of the COVID-19 pandemic. Specifically, for the increase in COVID-19 cases and deaths, we consider the growth rate of the announced cumulative number of confirmed COVID-19-positive cases per million and country and the growth rate of the announced deaths associated or caused by COVID-19 per million and country, respectively. However, this perspective assumes a linear progression of the pandemic.

In fact, as graphically visible in Fig. 1, the spread of COVID-19 can be separated into two phases: exponential growth of cases and deaths corresponding to initial global outbreaks, followed by logistic progression due to mitigated infection rates as global government responses unfold (De Silva et al. 2012). Epidemiological standard models can be used to address this nonlinearity. Comparing the performance of standard models for fast epidemics, Ma (2020) shows that the *susceptible-infectious-recovered model (SIR)*, first published by Hethcote (1989), provides a robust estimate for the spread of a disease exhibiting a pattern similar to COVID-19. SIR assumes the immunity of recovered individuals and incorporates the number of susceptible individuals. We calculate the SIR growth rate following Ma (2020) and Furtado (2021) as

3 $$\begin{gathered} \frac{{{\text{dS}}\left( {\text{t}} \right)}}{{{\text{dt}}}} = { } - \frac{{\upbeta }}{{\text{N}}}{\text{I}}\left( {\text{t}} \right){\text{S}}\left( {\text{t}} \right) \hfill \\ \frac{{{\text{dI}}\left( {\text{t}} \right)}}{{{\text{dt}}}} = { }\frac{{\upbeta }}{{\text{N}}}{\text{I}}\left( {\text{t}} \right){\text{S}}\left( {\text{t}} \right) - {\text{yI}}\left( {\text{t}} \right) \hfill \\ \frac{{{\text{dR}}\left( {\text{t}} \right)}}{{{\text{dt}}}} = {\text{yI}}\left( {\text{t}} \right) \hfill \\ \end{gathered}$$

where *S* is the share of susceptible individuals, *I* is the share of infectious individuals, and *R* is the share of recovered individuals. *ß* is the transmission rate per infectious individual, and *y* is the recovery rate. With the total number of individuals kept constant as the sum of *S*, *I*, and *R*, the expected growth rate is calculated as *λ* = *ß* – *y.* Descriptive statistics provide a mean of 4.531%. We recalculate our regression models, substituting our measures for the spread of the disease, i.e., CASES_c,t,_ by the SIR growth rate. Untabulated results remain congruent within all our regression models, indicating no distortion due to an imprecise, exponential fit of our COVID-19 proxies.

## Robustness tests

### Alternative benchmarks for the measurement of abnormal returns

Clearly, our study covers a worldwide economic crises that, without precedent, can be expected to systematically affect almost all sectors and firms worldwide. Thus, it is particularly important to find a benchmark for a firm’s returns that does not ignore the overarching effects of the crisis and is free of systematic influences. Thus, we employ three alternative approaches for the calculation of abnormal stock returns.

First, we recalculate our models using the daily average market return of the entire US market instead of focusing on the S&P 500. Therefore, we obtain the daily index prices of the Dow Jones U.S. Total Stock Market Index for our observation period. The index measures all US equity issues with available prices and covers 4224 firms and ten sectors. Similar to our main regression, we define the market adjustment of raw returns as $AR_{t}^{i} = R_{t}^{i} - E\left( {R_{t}^{i} } \right)$, where $R_{t}^{i}$ represents the daily return for firm *i* on day *t*. We estimate the firm’s expected return as $E(R_{t}^{i} ) = \alpha_{i} + b_{i} E(R_{t}^{market} )$, with $R_{t}^{market} = (P_{t}^{market} - P_{t - 1}^{market} )/P_{t - 1}^{market}$, where $P_{t}^{market}$ now represents the Dow Jones US. Total Stock Market Index closing price on day *t*. As all estimates are statistically indistinguishable from one another, evidence is provided for our main model’s results.

Second, we apply a portfolio-based approach to incorporate firm-specific risk instead of simply observing the daily market average. Specifically, following Fama and French (1995) and Kothari and Warner (2004), we compute a firm’s abnormal returns by adjusting the total returns for factors that have been found to explain cross-sectional differences in stock returns, i.e., a firm’s market capitalization and its price-to-book ratio. Similar to Brav et al. (2000), we form a set of floating portfolios of firms, with each portfolio expressing a distinct range of firm-size, i.e., a firm’s capitalization. Within each portfolio, we rank the firms by their price-to-book ratio. Based on our ranking values, we define a weighting factor $w_{j}$ for each ranking position. Total returns $R_{t}^{i}$ are then adjusted by the weighted total returns with varying values for each observation day: $AR_{t}^{i} = R_{t}^{i} - R_{t}^{i, port}$. Following Sul et al., (2014), we calculate $R_{t}^{i}$ as the natural logarithm of total returns plus one:$R_{t}^{i} = ln\left( {R_{t}^{i} + 1} \right)$. ${\varvec{R}}_{{\varvec{t}}}^{{{\varvec{i}},\user2{ port}}}$ therefore, can be defined as $\frac{1}{n}\sum\nolimits_{j = 1}^{n} {w_{j} \left( {R_{t}^{i} } \right)}$, where the sum of weights in each portfolio equals 1 ($\sum\nolimits_{j = 1}^{n} {w_{j} = 1}$). All results remain unchanged in direction and show insignificant divergences in magnitude.

### Aggregation of country-specific data on firm level

Our research design employs daily firm-country specific data. This approach carries important benefits that help to get deeper insights into the role of country-specific government responses when analyzing the pandemic’s impact on firms. Specifically, for each firm, we consider all countries worldwide that contribute to the firm’s revenues within our observation period. As a result, we split each firm into a set of *pseudo-subsidiaries*, with each subsidiary to solely reflect the effects of government responses of a single, distinct country on the firm’s stock prices over time.

In addition, the approach includes several country-specific control variables, e.g., media-sentiment variables, that continuously measure the spread and tone of pandemic-related news in a country. Following prior research that we discussed beforehand, they may be considered important drivers of investor sentiment.

However, a straightforward way of analyzing our dataset is to aggregate all data at the firm level. Specifically, on firm level, we weight a country’s daily COVID-19 related case and death numbers with its intensity of government responses. The result is a global variable for the overall strength of COVID-19 government policies for each firm. In this approach, again, all countries that contribute to the firm's sales revenues, are included. This calculation is blurred since it averages the country-specific government responses and the COVID-19 measures worldwide. However, it may still reinforce the robustness of our research design. We recalculate our regression models, using the aggregated data on firm level. Results remain unaffected and show similar magnitudes and directions.

### Further robustness tests

We perform further robustness tests to validate the results of our regression models. First, we include country-fixed effects to control for country-level heterogeneity. Again, results remain unchanged in direction and magnitude. Second, to control for cross-effects indicating that abnormal stock returns influence government responses, we repeat all regressions using RESPONSE_c,t_ as the dependent variable. We do not find significant results, supporting our main findings. Third, we conduct an in-time placebo test using placebo time windows to ensure that our regression results are not driven by our research design (e.g., Conley and Taber 2011; Hahn and Shi 2017). We run our main regression model shifting all independent variables back and forth 10, 15 and 30 trading days, respectively, holding our dependent variable, abnormal returns, constant. We fail to find any significant results, suggesting that, assuming no treatment effects, there is no evidence of random or systematic errors due to a weak model design. Forth, the reported results remain stable when conducting random effect regressions. Fifth, we use altering data frequencies to assure that our results are not sensitive to the daily-data approach. We aggregate all variables both weekly and monthly and rerun our main regressions. Results do not reveal divergences. Sixth, we recalculate our analysis substituting our dependent variable, abnormal returns, with raw returns. Results follow similar patterns throughout all models.

## Conclusion

In this paper, we analyze the role of worldwide government efforts to contain the spread and the economic consequences of the COVID-19 pandemic in shaping investor sentiment and stock market reactions. We explore the impact of government responses in the three fields of containment and closure, health system policies, and economic support of 180 countries on the relationship between growth rates of COVID-19 cases and deaths and firm-specific S&P 500 abnormal stock returns in a period from 1st January 2020 to 15th March 2021. We further investigate weather investor’s behavior is sensitive to a firm’s revenue exposure to COVID-19. We employ both an exponential growth model and an epidemiological standard model to account for the different stages of the pandemic and to address the nonlinear spread of the disease.

In contrast to previous studies, we find that deaths mainly drive stock returns during the COVID-19 pandemic. Undifferentiated, the entirety of government responses mitigates the decline of stock prices as market participants appreciate governments’ efforts to contain the consequences of the pandemic. Split by the different scopes of government responses, governments may mitigate negative market impacts caused by the pandemic by taking actions in the field of containment and closure, e.g., by school closings, workplace closings, closings of public transport or issuing stay-at-home orders. Similar effects stem from government’s economic support. Government support of a country’s health system provokes further declines in abnormal stock returns.

Analyzing the moderation of a firm’s revenue exposure to COVID-19, the mitigating effect of the entirety of government responses is even stronger for firms that are highly exposed to COVID-19 on the sales side. Differentiated by scope, this holds true for the field of government economic support. Containment and closure policies do not seem strong enough for investors to adjust their pessimistic views on market development. Hence, we find no mitigation of stock price declines due to growing COVID-19 case numbers. Contrary to our initial findings, government support of health systems is rated more positively by investors when a firm is more severely affected by the pandemic on the sales side. The additional positive stock market effect for firms that are highly exposed to the pandemic even reverses the initial negative impact. These results remain unchanged when the spread of COVID-19 is estimated using an epidemiological standard model, i.e., the SIR, to account for the pandemic’s nonlinear course.

Our findings contribute to both practice and research. First, firms that become aware of both the pandemic’s impact on investor sentiment and the moderating role of government responses may be able to anticipate and strategically manage investor relations. Regarding investors’ reactions to a firm’s country-specific revenue exposure to COVID-19, firms should aim to redirect and adjust the content and scope of their communications with investors. They may also aim for a dialog with governments to encourage aid in firm-relevant fields. Second, our results are relevant for government regulators debating the economic costs and benefits of government responses to pandemics, since no case of such extent is yet known, and reliable data concerning the consequences of government interventions for medical crises are rare. Third, our study expands the knowledge about investor sentiment as a driver of stock prices during external shocks followed by crisis situations.

As with all studies, our study is limited in several ways and, as such, paves the way for future research. As we derive investor sentiment indirectly by measuring abnormal stock market reactions, qualitative studies will be necessary to more deeply investigate investors’ behavior during the COVID-19 pandemic. Although investor reactions are clearly visible, the psychological motives as well as the strength, composition, and persistence of investor reactions remain unclear and require further investigation. We analyze whether investor’s perceptions of government responses to the COVID-19 pandemic are affected by the share of sales revenues that is threatened by the pandemic in each country. However, this approach compromises several weaknesses since the linkage between the case and death development and sales revenue may not be linear. We encourage further research to build on this bias and seek for more accurate approaches to reflect the risk of losing sales revenues during pandemics. Moreover, we do not analyze whether firm characteristics other than country-specific sales revenues may mitigate or reinforce stock market reactions. For example, the exposure of worldwide supply chains may cause different effects on the stock market and, thus, should be a matter of further research. As our dataset provides firm-country-specific data, further studies may cluster countries and reveal the role of policies and measures among geographic regions or political and economical unions.

## Data Availability

Data are available from commercial databases and public sources identified in the paper.

## Appendix 1

Country-specific S&P 500 firm allocation

**Table:**

No	Country	Number of Firms	Share of S&P 500	No	Country	Number of Firms	Share of S&P 500
1	Afghanistan	154	30.14	51	El Salvador	195	38.16
2	Albania	190	37.18	52	Eritrea	44	8.61
3	Algeria	251	49.12	53	Estonia	249	48.73
4	Andorra	9	1.76	54	Ethiopia	234	45.79
5	Angola	237	46.38	55	Faroe Islands	9	1.76
6	Argentina	289	56.56	56	Fiji	17	3.33
7	Aruba	21	4.11	57	Finland	308	60.27
8	Australia	312	61.06	58	France	331	64.77
9	Austria	316	61.84	59	Gabon	136	26.61
10	Azerbaijan	223	43.64	60	Gambia	2	0.39
11	Bahamas	103	20.16	61	Georgia	198	38.75
12	Bahrain	220	43.05	62	Germany	334	65.36
13	Bangladesh	283	55.38	63	Ghana	226	44.23
14	Barbados	40	7.83	64	Greece	303	59.30
15	Belarus	278	54.4	65	Guam	20	3.91
16	Belgium	320	62.62	66	Guatemala	249	48.73
17	Belize	12	2.35	67	Guinea	114	22.31
18	Benin	123	24.07	68	Guyana	45	8.81
19	Bermuda	47	9.2	69	Haiti	64	12.52
20	Bhutan	13	2.54	70	Honduras	184	36.01
21	Bolivia	232	45.4	71	Hungary	310	60.67
22	Bosn. & Herzeg	206	40.31	72	Iceland	226	44.23
23	Botswana	149	29.16	73	India	310	60.67
24	Brazil	305	59.69	74	Indonesia	279	54.60
25	Brunei Darussalam	102	19.96	75	Iran	269	52.64
26	Bulgaria	284	55.58	76	Iraq	264	51.66
27	Burkina Faso	135	26.42	77	Ireland	322	63.01
28	Burundi	12	2.35	78	Israel	275	53.82
29	Cabo Verde	2	0.39	79	Italy	325	63.60
30	Cambodia	194	37.96	80	Jamaica	131	25.64
31	Cameroon	207	40.51	81	Japan	315	61.64
32	Canada	350	68.49	82	Jordan	222	43.44
33	Cent. Afr. Rep	2	0.39	83	Kazakhstan	262	51.27
34	Chad	98	19.18	84	Kenya	237	46.38
35	Chile	287	56.16	85	Kiribati	1	0.20
36	Colombia	288	56.36	86	Kuwait	258	50.49
37	Congo	92	18	87	Kyrgyzstan	73	14.29
38	Costa Rica	244	47.75	88	Laos	168	32.88
39	Cote d'Ivoire	222	43.44	89	Latvia	260	50.88
40	Croatia	283	55.38	90	Lebanon	232	45.40
41	Cuba	246	48.14	91	Lesotho	5	0.98
42	Cyprus	229	44.81	92	Liberia	7	1.37
43	Czech Republic	315	61.64	93	Libya	218	42.66
44	Denmark	312	61.06	94	Liechtenstein	93	18.20
45	Djibouti	10	1.96	95	Lithuania	280	54.79
46	Dominica	4	0.78	96	Luxembourg	293	57.34
47	Dom. Republic	245	47.95	97	Madagascar	122	23.87
48	DR of the Congo	216	42.27	98	Mainland China	324	63.41
49	Ecuador	262	51.27	99	Malawi	55	10.76
50	Egypt	272	53.23	100	Malaysia	281	54.99
101	Mali	141	27.59	151	Sudan	187	36.59
102	Malta	193	37.77	152	Suriname	27	5.28
103	Mauritania	49	9.59	153	Swaziland	24	4.70
104	Mauritius	124	24.27	154	Sweden	315	61.64
105	Mexico	317	62.04	155	Switzerland	317	62.04
106	Moldova	166	32.49	156	Syria	232	45.40
107	Monaco	101	19.77	157	Tajikistan	69	13.50
108	Mongolia	115	22.50	158	Tanzania	225	44.03
109	Morocco	247	48.34	159	Thailand	298	58.32
110	Mozambique	135	26.42	160	Timor-Leste	10	1.96
111	Myanmar	254	49.71	161	Togo	37	7.24
112	Namibia	120	23.48	162	Tonga	2	0.39
113	Nepal	209	40.90	163	Trinidad and Tobago	171	33.46
114	Netherlands	326	63.80	164	Tunisia	201	39.33
115	New Zealand	274	53.62	165	Turkey	277	54.21
116	Nicaragua	104	20.35	166	Turkmenistan	226	44.23
117	Niger	110	21.53	167	Uganda	200	39.14
118	Nigeria	263	51.47	168	Ukraine	299	58.51
119	Norway	309	60.47	169	United Arab Emirates	276	54.01
120	Oman	240	46.97	170	United Kingdom	355	69.47
121	Pakistan	266	52.05	171	United States	501	98.04
122	Palestine	126	24.66	172	Uruguay	248	48.53
123	Panama	242	47.36	173	US Virgin Islands	31	6.07
124	Papua New Guinea	167	32.68	174	Uzbekistan	233	45.60
125	Paraguay	233	45.60	175	Vanuatu	2	0.39
126	Peru	281	54.99	176	Venezuela	254	49.71
127	Philippines	273	53.42	177	Vietnam	284	55.58
128	Poland	322	63.01	178	Yemen	187	36.59
129	Portugal	310	60.67	179	Zambia	166	32.49
130	Puerto Rico	254	49.71	180	Zimbabwe	155	30.33
131	Qatar	262	51.27				
132	South Korea	308	60.27				
133	Romania	310	60.67				
134	Russian Federation	318	62.23				
135	Rwanda	88	17.22				
136	San Marino	3	0.59				
137	Saudi Arabia	274	53.62				
138	Senegal	168	32.88				
139	Serbia	269	52.64				
140	Seychelles	1	0.20				
141	Sierra Leone	16	3.13				
142	Singapore	304	59.49				
143	Slovakia	298	58.32				
144	Slovenia	279	54.60				
145	Solomon Islands	7	1.37				
146	Somalia	48	9.39				
147	South Africa	268	52.45				
148	South Sudan	7	1.37				
149	Spain	327	63.99				
150	Sri Lanka	256	50.10				

This table provides the distribution of included firms by country. A firm is assigned to a country if the country contributes to the firm’s sales revenues. The share of S&P 500 firms that exhibit revenue in the specific country is displayed in the right column. We use country-specific sales revenue data for the compounding S&P 500 firms for 2019 from the *FactSet Geographic Revenue (GeoRev)* Database. We only include values with a certainty score of 70 or above, as provided by *GeoRev*. The certainty score is based on source metadata and ranges from 1 (low certainty) to 80 (declared value).

## Appendix 2

**Table:**

List of indicator descriptions and scale codings		
Name	Description	Coding
Containment and closure policies		
School closing	Record closings of schools and universities	0—no measures 1—recommend closing or all schools open with alterations resulting in significant differences compared to non-Covid-19 operations 2—require closing (only some levels or categories, e.g., just high school or just public schools) 3—require closing all levels Blank—no data
Workplace closing	Record closings of workplaces	0—no measures 1—recommend closing (or recommend work from home) or all businesses open with alterations resulting in significant differences compared to non-Covid-19 operation 2—require closing (or work from home) for some sectors or categories of workers 3—require closing (or work from home) for all-but-essential workplaces (e.g., grocery stores, doctors) Blank—no data
Cancel public events	Record canceling public events	0—no measures 1—recommend canceling 2—require canceling Blank—no data
Restrictions on gatherings	Record limits on gatherings	0—no restrictions 1—restrictions on very large gatherings (above 1000 people) 2—restrictions on gatherings between 101 and 1000 people 3—restrictions on gatherings between 11 and 100 people 4—restrictions on gatherings of 10 people or less Blank—no data
Close public transport	Record closing of public transport	0—no measures 1—recommend closing (or significantly reducing volume/route/means of transport available) 2—require closing (or prohibit most citizens from using it) Blank—no data
Stay at home requirements	Record orders to "shelter-in-place" and otherwise confine to the home	0—no measures 1—recommend not leaving house 2—require not leaving house with exceptions for daily exercise, grocery shopping, and 'essential' trips 3—require not leaving house with minimal exceptions (e.g., allowed to leave once a week, or only one person can leave at a time, etc.) Blank—no data
Restrictions on internal movement	Record restrictions on internal movement between cities/regions	0—no measures 1—recommend no travel between regions/cities 2—internal movement restrictions in place Blank—no data
International travel controls	Record restrictions on international travel Note: this records policy for foreign travelers, not citizens	0—no restrictions 1—screening arrivals 2—quarantine arrivals from some or all regions 3—ban arrivals from some regions 4—ban on all regions or total border closure Blank—no data
Health system policies		
Public information campaigns	Record presence of public info campaigns	0—no Covid-19 public information campaign 1—public officials urging caution about Covid-19 2—coordinated public information campaign (e.g., across traditional and social media) Blank—no data
Testing policy	Record government policy on who has access to testing Note: this records policies about testing for current infection (PCR tests) not testing for immunity (antibody test)	0—no testing policy 1—only those who both (a) have symptoms AND (b) meet specific criteria (e.g., key workers, admitted to hospital, came into contact with a known case, returned from overseas) 2—testing of anyone showing Covid-19 symptoms 3—open public testing (e.g., "drive through" testing available to asymptomatic people) Blank—no data
Contact tracing	Record government policy on contact tracing after a positive diagnosis Note: policies that would identify all people potentially exposed to Covid-19; voluntary bluetooth apps are unlikely to achieve this	0—no contact tracing 1—limited contact tracing; not done for all cases 2—comprehensive contact tracing; done for all identified cases
Facial coverings	Record policies on the use of facial coverings outside the home	0—No policy 1—Recommended 2—Required in some specified shared/public spaces outside the home with other people present, or some situations when social distancing not possible 3—Required in all shared/public spaces outside the home with other people present or all situations when social distancing not possible 4—Required outside the home at all times regardless of location or presence of other people
Vaccination policy	Record policies for vaccine delivery for different groups	0—No availability 1—Availability for ONE of following: key workers/clinically vulnerable groups (non-elderly)/elderly groups 2—Availability for TWO of following: key workers/clinically vulnerable groups (non-elderly)/elderly groups 3—Availability for ALL of following: key workers/clinically vulnerable groups (non-elderly)/elderly groups 4—Availability for all three plus partial additional availability (select broad groups/ages) 5—Universal availability
Protection of elderly people	Record policies for protecting elderly people (as defined locally) in long term care facilities (LTCF) and/or community and home settings	0—no measures 1—Recommended isolation, hygiene, and visitor restriction measures in LTCFs and/or elderly people to stay at home 2—Narrow restrictions for isolation, hygiene in LTCFs, some limitations on external visitors and/or restrictions protecting elderly people at home 3—Extensive restrictions for isolation and hygiene in LTCFs, all nonessential external visitors prohibited, and/or all elderly people required to stay at home and not leave the home with minimal exceptions, and receive no external visitors Blank—no data
Economic support		
Income support (for households)	Record if the government is providing direct cash payments to people who lose their jobs or cannot work Note: only includes payments to firms if explicitly linked to payroll/salaries	0—no income support 1—government is replacing less than 50% of lost salary (or if a flat sum, it is less than 50% of median salary) 2—government is replacing 50% or more of lost salary (or if a flat sum, it is greater than 50% of median salary) Blank—no data
Debt/contract relief (for households)	Record if the government is freezing financial obligations for households (e.g., stopping loan repayments, preventing services like water from being stopped, or banning evictions)	0—no debt/contract relief 1—narrow relief, specific to one kind of contract 2—broad debt/contract relief

This table provides the descriptions and scale codings of all indicators composing the indices for our three policy fields, i.e., containment and closure index, health system index, and economic support index. To create the indices, subindices are calculated for all indicators to normalize each indicator to an equally spaced scale between 0 and 100. The three indices are then calculated as simple averages of the normalized individual subindices. This table is provided by *Oxford University’s Government Response Tracker (OxCGRT)*

## Appendix 3

Variable definitions

**Table:**

Variables	Definition	Level	Frequency	Data source
Dependent variable				
AR	Adjusted abnormal logarithmic returns based on a single-index market model. Expected returns are estimated with market betas using the firm’s daily stock returns, and the S&P 500 index returns over an estimation window of 120 trading days, starting one day prior to the measurement day [− 1; − 121]	Firm	Daily	Refinitiv Datastream
Variables of interest				
CASES	Daily growth rate of the announced cumulative number of confirmed COVID-19-positive cases per million and country	Country	Daily	European Centre for Disease Prevention and Control (ECDC)
DEATHS	Daily growth rate of the announced deaths associated or caused by COVID-19 per million and country	Country	Daily	ECDC
EXPOSURE	Weighted country-specific annualized sales revenues [REVENUEi,c,t] with the country’s daily growth rates of COVID-19 cases [CASESc,t], and deaths [DEATHSc,t] per million, respectively. The variable is standardized using z-scores and normalized to a scale from 0 to 100, where higher values indicate a larger firm-specific revenue exposure to COVID-19	Firm-Country	Daily	ECDC
CONTAINMENT_CLOSURE	Index measure for the strictness of a country’s COVID-19 policies to contain the spread of the disease. Composing indicators: school closing; workplace closing; cancel public events; restrictions on gatherings; close public transport; stay at home requirements; restrictions on international movement; international travel controls	Country	Daily	Oxford University’s Government Response Tracker (OxCGRT)
HEALTH_SYSTEM	Index measure for a country’s efforts to strengthen its health systems. Composing indicators: public information campaigns; testing policy; contact tracing; facial covering; vaccination policy; protection of elderly people	Country	Daily	OxCGRT
ECON_SUPPORT	Index measure for the extent of a country’s economic support. Composing indicators: income support; debt and contract relief	Country	Daily	OxCGRT
GOV_RESPONSE	Index measure for a country’s summarized countermeasures. All indicators included	Country	Daily	OxCGRT
Control variables				
REVENUE	Estimated percentage of revenue a firm derives from an associated country, measured annually. We only include values with a certainty score of 70 or above, as provided by FatSet GeoRev. The certainty score is based on source metadata and ranges from 1 (low certainty) to 80 (declared value)	Firm-Country	Annually	FactSet Revere Geographic Exposure (GeoRev)
VOLATILITY	Stock volatility of daily raw returns	Firm	Daily	Refinitiv Datastream
FOLLOWING	Natural logarithm of 1 plus the number of analysts following a firm	Firm	Annually	I/B/E/S
INSTITUTIONAL	Percentage of shares held by institutional investors	Firm	Annually	I/B/E/S
SIZE	Natural logarithm of 1 plus a firm’s total sales	Firm	Annually	Refinitiv Datastream
PTB	A firm’s book value per share over its latest closing stock price	Firm	Daily	Refinitiv Datastream
BOARD	The number of a firm’s board members	Firm	Quarterly	Refinitiv Datastream
LEVERAGE	A firm’s book value debt over its total assets	Firm	Yearly	Refinitiv Datastream
ROA	A firm’s operating income before depreciation over total assets	Firm	Quarterly	Refinitiv Datastream
ESG	Overall firm score based on the self-reported information in the environmental, social, and corporate governance pillars	Firm	Weekly	Refinitiv Datastream
MEDIA_COVERAGE	Daily percentage of all news agencies in a country that cover the topic of COVID-19	Country	Daily	Ravenpack Coronavirus Media Monitor
MEDIA_HYPE	Daily percentage of news reports that are covering the topic COVID-19 in a country	Country	Daily	Ravenpack Coronavirus Media Monitor
MEDIA_SENTIMENT	Daily average level of sentiment that those news reports express towards a firm, that mention both the COVID-19 pandemic and the firm in the report. Measured as the daily average of the difference between the number of positive and negative news reports fulfilling these criteria. A report’s sentiment is determined by systematically matching stories usually categorized by financial experts as having a positive or negative financial or economic impact	Country	Daily	Ravenpack Coronavirus Media Monitor

This table provides variable definitions for all variables used in our main regressions

## Footnotes

1 In the original dataset, Hale et al. (2020) present four indices to give an overall impression of government activity. However, these indices cover a mix of indicators from all three policy groups. To accurately reflect government responses of each policy group, we calculate our own indices, solely including indicators of the assigned group of policies. * We also control for country-fixed (Country FE) effects as part of our robustness tests. Please see Sect. 5.

2 Since the COVID-19 pandemic most likely causes widespread systematic effects across firms and sectors worldwide, it is imperative to employ alternative and neutral benchmarks for calculating abnormal returns. Thus, we repeat our analysis using a portfolio-based approach to calculate abnormal returns based on firm-specific risk. In addition, we recalculate our models using the daily average market return of the entire US market instead of focusing the S&P 500. Both analyses are presented in section five (Robustness Tests).

3 "ESG scores by Refinitiv have been used (or referenced) in more than 1200 academic articles over the past 15 years. Moreover, Refinitiv ESG data are used by major asset managers, such as BlackRock, to manage ESG investment risks. Refinitiv ESG data are also referenced in an ESG white paper featured at the World Economic Forum in 2019 (WEF 2019), and analyzed as one of the three key ratings providers in a recent OECD report." (Boffo and Paralano 2020).

4 Source: *RefinitivDatastream.*
